# Innovations on a shoestring: a study of a collaborative community-based Aboriginal mental health service model in rural Canada

**DOI:** 10.1186/1752-4458-3-27

**Published:** 2009-12-17

**Authors:** Marion A Maar, Barbara Erskine, Lorrilee McGregor, Tricia L Larose, Mariette E Sutherland, Douglas Graham, Marjory Shawande, Tammy Gordon

**Affiliations:** 1 Northern Ontario School of Medicine, Laurentian University, 935 Ramsey Lake Road, Sudbury, ON, P3E 2C6, Canada; 2 Noojmowin Teg Health Centre, Postal Bag 2002, Hwy 540, 48 Hillside Rd., Aundeck Omni Kaning, Little Current, Ontario, P0P 1K0, Canada; 3 Community Based Research, General Delivery Birch Island, P0P 1A0, Canada; 4 Laurentian University, School of Rural and Northern Health, 935 Ramsey Lake Road, Sudbury, ON, P3E 2C6, Canada; 5 Mnaamodzawin Health Services Inc, Postal Bag 2002, Hwy 540, 48 Hillside Rd., Aundeck Omni Kaning, Little Current, Ontario, P0P 1K0, Canada

## Abstract

**Background:**

Collaborative, culturally safe services that integrate clinical approaches with traditional Aboriginal healing have been hailed as promising approaches to ameliorate the high rates of mental health problems in Aboriginal communities in Canada. Overcoming significant financial and human resources barriers, a mental health team in northern Ontario is beginning to realize this ideal. We studied the strategies, strengths and challenges related to collaborative Aboriginal mental health care.

**Methods:**

A participatory action research approach was employed to evaluate the Knaw Chi Ge Win services and their place in the broader mental health system. Qualitative methods were used as the primary source of data collection and included document review, ethnographic interviews with 15 providers and 23 clients; and 3 focus groups with community workers and managers.

**Results:**

The Knaw Chi Ge Win model is an innovative, community-based Aboriginal mental health care model that has led to various improvements in care in a challenging rural, high needs environment. Formal opportunities to share information, shared protocols and ongoing education support this model of collaborative care. Positive outcomes associated with this model include improved quality of care, cultural safety, and integration of traditional Aboriginal healing with clinical approaches. Ongoing challenges include chronic lack of resources, health information and the still cursory understanding of Aboriginal healing and outcomes.

**Conclusions:**

This model can serve to inform collaborative care in other rural and Indigenous mental health systems. Further research into traditional Aboriginal approaches to mental health is needed to continue advances in collaborative practice in a clinical setting.

## Background

Research shows a significantly higher prevalence of mental illness, particularly depression, suicide and addictions in Aboriginal communities when compared with the broader Canadian population[1]. Interventions to improve Aboriginal mental health must be informed, not only by the people within these communities, but also by the colonial history that has contributed to this poor health profile. Multi-generational marginalization, power disparities and governmental assimilation practices and in particular the residential school systems subjected generations of Aboriginal people in Canada to physical, mental, spiritual and sexual abuse and eroded family relationships; furthermore, loss of language, land, culture and traditional economies have had devastating impacts on the social determinants of health [2-4]. This connection between colonial forces and mental illness and addictions is supported by an increasing body of literature [3,5-10]. Research in other countries with a colonial history, such as New Zealand, Australia and the USA show very similar patterns of mental health issues among Indigenous populations [11-13].

Despite high rates of mental health problems, there is no national Aboriginal mental health strategy in Canada. A few fragmented national services do exist, accessible only to a relatively small proportion of Aboriginal people, dependant on their legal status and place of residence. These services include community-based paraprofessional addictions services, regional treatment centres and short term clinical treatment for crisis intervention consisting of coverage for up to 10 treatment sessions [14,15]. These programs do not provide the range and quality of services required to address the complexity of mental health problems in Aboriginal communities; consequently there are serious gaps in services. Crises, particularly in rural and remote communities, are not uncommon. In turn, 'outside' interventions may be parachuted into communities, following local emergencies such as community violence or youth suicide waves. However, they are often rejected by Aboriginal people, because these services are essentially value laden, lack cultural safety and *"have shallow histories in aboriginal communities" *[16]. Clearly, a community-driven approach is essential to achieve community acceptance and to organize the scarce resources in a way that supports the development of local systems of care[17].

Collaborative and culturally competent services that integrate clinical as well as traditional Aboriginal healing services have been identified as promising approaches for Aboriginal mental health services for some time [18]. Yet, barriers to realize this ideal are substantial. Recruiting mental health professionals to rural and remote areas where many Aboriginal communities are located is difficult and without a national strategy, funds are lacking. Furthermore, a commitment to interprofessional education is a prerequisite for an integrated approach. Nevertheless, over the past decade First Nations (Indigenous communities) in the Manitoulin District in Northern Ontario, Canada have created an integrated community-based mental health services system by successfully pooling resources and developing collaborative programs.

Prior to this research, anecdotal evidence suggested that this new and evolving collaborative approach to Aboriginal community mental health showed indications of positive outcomes such as: (1) improved access to a range of mental health services in a rural environment, (2) increasing continuity of care, (3) improved cultural safety, (4) integration of clinical and traditional Aboriginal services, and (5) a stable mental health team with low attrition rates. Our study was designed to go beyond the anecdotal evidence to identify critical components and outcomes of this model. We focused on three research questions: How is collaborative mental health care provided in this northern Aboriginal context? What strategies support interdisciplinary collaboration and service integration? And, what are the strengths and challenges related to this model?

## Methods

### Participatory Action Research

We used a participatory action approach; as such, the project was designed to be highly collaborative, relevant and empowering for stakeholders[19]. A steering committee consisting of Aboriginal elders, community members and local decision makers was formed to oversee the research process. Academic researchers, community-based researchers, and local stakeholders collaborated closely during all phases of the research. For example, the steering committee collaborated on research questions, advised on implementation of the project, provided a cultural perspective for the interpretation of findings and reviewed the final research report. The Knaw Chi Ge Win Team (i.e. the core mental health team) provided technical advice and valuable background information that informed our methods. To address the research questions, we used qualitative research methods to analyze and triangulate multiple sources of information, including program documents as well as ethnographic interviews and focus groups with clients and service providers.

### Document Review

We reviewed annual mental health program reports, service data, and past program proposals from 2004 to 2007. These documents served to substantiate and triangulate information obtained from interviews and focus groups.

### Ethnographic Interviews and Focus Groups with Service Providers

The key informant interviews were conducted with the Knaw Chi Ge Win team, visiting mental health consultants, therapists and social workers in the mainstream service system, and tribal police. Focus group participants included First Nations community administrators and community-based health workers. A total of 31 key informants participated (see Table 1). Interviews and focus groups were conducted and audio recorded by the principal investigator (MAM) and community-based researchers (LM and MES).

**Table 1:** Interview and Focus Group Breakdown

METHOD	SCHEDULED LENGTH OF SESSION	GROUP	# OF PARTICIPANTS
Community Service Provider Focus Groups	1.5 hours	Band Managers	5
		Mental health workers	4
		Community health representatives & nurses	7

Provider Interviews	1 hour	Mental Health Team members, social service and related providers	15

Client Interviews	30 minutes	Clients	23

**Total number of participants**			**54**

### Ethnographic Interviews with Aboriginal Clients

A client interview protocol was developed that ensured safe, ethical and standardized client interviews. Clients who received services in the past two years were eligible to participate in the research. Potential participants were randomly contacted by administrative staff and over 85% of those invited agreed to participate. Participants were offered a small monetary compensation for their time and travel expenses. Ethnographic interviews were conducted with clients, documenting their experience with the mental health services until thematic saturation was reached. Questions were focused on the perceived level of care and cultural safety, access and barriers to care as well as overall satisfaction with services. 23 client interviews were conducted by experienced community-based Aboriginal researchers (LM and MES). Sessions were audio recorded. The Knaw Chi Ge Win team was available to provide follow up counseling services if necessary.

### Method of Analysis

Interviewers completed summary sheets for each client interview and identified key themes in client responses. The audio recordings of interviews and focus group were transcribed by a single research assistant (TLL) to ensure consistency. Transcripts were analyzed using the qualitative analysis software NVivo 7 [20]. We conducted a thematic analysis of the experience of clients and providers with the local mental health services [21]. Emerging themes related to the central research questions and participants' experiences were identified and coded using *in vivo *coding when possible. The coding system was verified though "member checking" by comparing the identified themes with the interviewers' summary sheets. Discrepancies were discussed and resolved during follow up meetings. An interactive presentation of draft findings to the steering committee and the mental health team served as a further level of verification [22].

### Ethics

The First Nations in the Manitoulin District have made significant strides in building community research capacity and engaging in research partnerships with university-based researchers [23,24]. In agreement with the local research ethics guidelines, ethics review was obtained from the Manitoulin Aboriginal Research Review Committee and the research concept was approved by local First Nations leaders and health boards[25]. The four principles of OCAP [26], namely: First Nations *ownership, control, access *and *possession *of research conducted in their communities were adapted to fit the context of this study. With respect to *ownership *and *control*, our approach emphasized consensus in all aspects of the research process rather than a power relationship between community and university stakeholders. *Access *to research results was created in the form of research reports and community and staff presentation prior to wider publications. The stakeholders agreed that in order to maximize client and provider confidentiality, raw interview and focus group data was only to be held in the *possession *of the university based researchers and not at local organizations. The interpretation of OCAP in this study was strongly influenced by longstanding respect based relationship between the community stakeholders and the lead researchers.

## Results

### Overview of the Service Model

**Figure 1:**
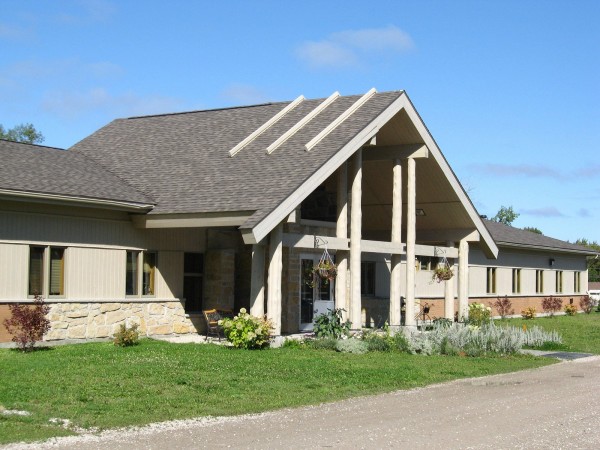
**The Share Office Building**.

**Figure 2:**
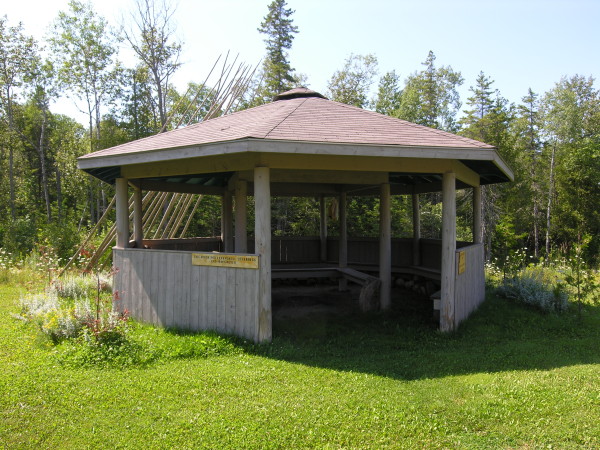
**Arbor used for Traditional Aboriginal Healing Services**.

The Knaw Chi Ge Win service system we studied is located on Manitoulin Island in Northern Ontario. Communities on this large rural island include seven First Nations communities. First Nations people account for over 1/3 of the approximately 13,000 local residents and the island's population density is 2.8 people per square kilometer[27]. Family physician services are available in several of the larger communities. Most specialized services however are located in the nearest urban centre, at a driving distance of 1.5 to 3 hours from island communities. Knaw Chi Ge Win services are coordinated by two regional Aboriginal health organizations. The first organization, Mnaamodzawin Health Services (MHS) is a regional provider of First Nations community health services. The second one, Noojmowin Teg Health Access Centre (NT) is a regional provider of interdisciplinary primary care services. Both organizations have a distinct emphasis on community-based Aboriginal approaches to care and share a home office (see Figure 1 and Figure 2). Service integration, such as common intake, case coordination and seamless services without waiting periods or administrative barriers was a common goal and was initiated with the formation of an interdisciplinary mental health care group, named the Knaw Chi Ge Win (New Beginnings) team.

### Knaw Chi Ge Win Services

The Knaw Chi Ge Win Team is a core of providers with expertise in psychology, mental health nursing, long-term care, social work and traditional Aboriginal medicine and healing. Clients can access services by referral or self-referral. In addition to the core services, visiting specialist consultant services are provided by psychiatrists and traditional Aboriginal healers, accessed by referral from the core team. Table 2 provides a list of the composition of staff and contract services. Mental health services are provided within an holistic Aboriginal framework that acknowledges the physical, mental, emotional, and spiritual aspects of health as well as historical, socioeconomic and cultural influences[28]. The core team's home office is centrally located within the region and services can be accessed at the main office, in community clinics located in seven First Nation communities, or through home visits. This model of service provision improves access to mental health services compared with a model where clients travel to a central location to receive services. It also increases privacy for clients. As one participant explained, clients often know their local community clinic staff personally and are uncomfortable accessing mental health services there: *"I always felt...walking in the building it's like [staff might say]: 'Oh, I wonder what's she in here for'." (Client #2)*.

**Table 2:** Composition of Core Team and Contract Consultants

POSITION	INTEGRATION LEVEL	FTE^**1 **^OR CONTRACT	EMPLOYER	FUNDING STREAM
Mental health program manager	Core team	1 FTE	MHS	Health Transfer ^2^

Psychologist	Core team	1 FTE	NT	Aboriginal Healing and Wellness Strategy^3^

Traditional coordinator	Core team	1 FTE	NT	Aboriginal Healing and Wellness Strategy

Mental health workers/clinicians	Core team	2 FTE	MHS	Health Transfer

Mental health nurse/case manager	Core team	1 FTE	MHS	Health Transfer

Psychiatrist	Secondary service	1 day per month Contract	Contract services	Health Transfer

Visiting Psychologist	Secondary service	2 days per month Contract	Contract services	Aboriginal Healing and Wellness Strategy

Traditional Healer	Secondary service	4-5 days per month Contract	Contract services	Aboriginal Healing and Wellness Strategy

1 fulltime equivalent

2 a federal initiative under FNIHB

3 a provincial initiative unique to Ontario

### Collaboration with Visiting Consultants

The services provided by the Knaw Chi Ge Win team are enhanced by integrated specialized services by visiting consultants, in particular psychiatric services and traditional healing services. There are no internal administrative barriers to access these consultant services, and clients are placed on a list for the next visit (waiting periods are up to one month) based on recommendation of team members. In order to encourage collaborative practice with the Knaw Chi Ge Win team, consultants are compensated based on an hourly rate rather than a fee for service structure. Psychiatric services are offered on a monthly basis and client care is shared with the team to ensure continuity of care and follow up after the consult. Team members attend sessions with their clients and regularly consult in person with the psychiatrist. Consultant notes are completed for primary care physicians in nearby clinics to further improve continuity of care.

To ensure that cultural protocols related to traditional healing were upheld, in depth consultations with communities were required to determine the method of compensation for the traditional Aboriginal healers. Today, the services of healers are also compensated under a consultant contract. The traditional coordinator is the Knaw Chi Ge Win team member who normally attends traditional healing sessions with clients and shares their care. The traditional coordinator has a background in both nursing as well as traditional approaches to healing and maintains client charts, monitors herbal medicines, and coordinates follow up such as clinical mental health and primary care. The traditional coordinator assesses, in collaboration with the healer, if clients require clinical mental health services and refers when necessary. Traditional services are in high demand by community members, as such, there is not sufficient time for the healer to review cases with mental health workers although this is perceived as desirable.

### Coordination with other Care Providers in the Region

For clients with chronic mental illnesses the visiting psychiatrist and the Knaw Chi Ge Win case manager collaborate with the primary care physician. The case manager updates physicians on treatments. Collaboration between other team members and physicians is limited, on an *ad hoc *basis, and often based on established personal relationships between providers. *Ad hoc *collaboration occurs usually when clients are admitted to emergency services. The collaboration with private practice therapists who provide short term counseling services under coverage by Health Canada's First Nations and Inuit Health Branch (FNIHB) crisis counseling program consist solely of referrals[14].

**Figure 3:**
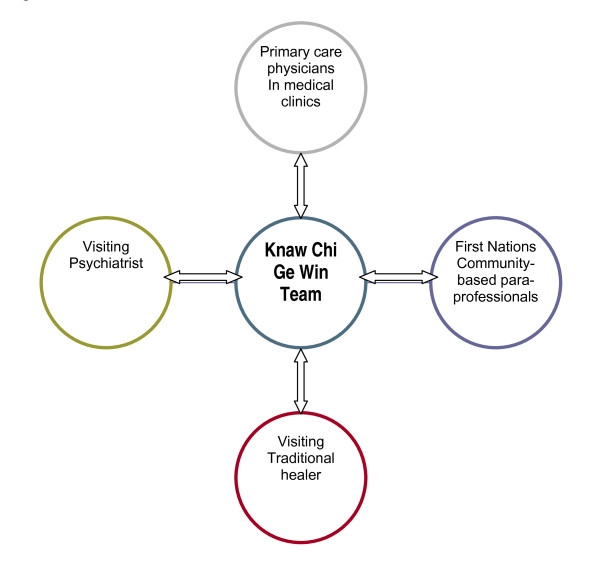
**Conceptual Map of the Shared Care Network of the Knaw Chi Ge Win Team**. (attached as separate files)

Collaboration with First Nations community-based paraprofessionals varies and depends on each worker's background, job expectations and, most importantly, the client's comfort with receiving care from their local mental health worker. Collaborative practice includes prevention program development as well as client services that support the daily management of clients with serious mental illness (SMI) and clients who access traditional medicine. An increased level of integration in direct client services is desired by many paraprofessional workers. Figure 3 provides a map of the major collaborative care relationships maintained by the Knaw Chi Ge Win team.

### Regional Planning and Networking

The Knaw Chi Ge Win team also focuses on the development of a mental health care system in the region. Formal networks, such as a district wide networking group bringing together addictions and mental health service providers, have been created. These networks also serve to connect Aboriginal and mainstream agencies to advance coordination of care. They also provide a forum for advocacy for Aboriginal mental health issues, and the establishment of working relationships with acute care providers and the social services sector. A regional strategic planning process for mental health has also been implemented.

### Strategies that Support Interprofessional Collaboration

To function well in interprofessional teams, health care professionals must overcome barriers such as professional rivalry and negative stereotypes and thus require specialized training [29-31]. Similarly, interprofessional collaboration within the Knaw Chi Ge Win team required planning and nurturing. The shared home office space is an important enabling component as it creates an environment where providers have frequent face-to-face contact. In addition, Knaw Chi Ge Win providers developed strategies and activities to support collaboration and facilitate a continuum of care. We describe these below.

### Weekly intake meetings

The core mental health team members review new client cases at weekly intake meetings and assign clients to the most suitable mental health provider(s) while peer feedback and support is strongly encouraged. (Clients are informed and provide written consent that their information is shared within the team). Services coordinated at that time may include mental health counseling, psychiatry services, traditional healing services and referrals to long-term care providers or nurse practitioners. This team approach to intake facilitates a holistic approach to care and a rapid response for clients who require urgent care and multiple services. Furthermore, new team members learn about the local mental health system, communities and Aboriginal culture, as well as common mental health issues and effective treatment approaches.

### Peer Supervision and Informal Case Consultation

Clinical supervision is a key component of mental health practice [32], but it poses challenges in a rural, multi-disciplinary environment with limited resources. Consequently, the Knaw Chi Ge Win team has opted for a peer supervision process, consisting of *ad hoc *informal case reviews and consultations with colleagues. To invite peer supervision, providers maintain an open door policy and informal consultations are quite common among the Knaw Chi Ge Win team. Workers have recently initiated client case presentations as a tool for peer supervision and interprofessional education. These presentations demonstrate the response of various professionals to difficult client situations. Providers report positive outcomes and the following quote shows high levels of satisfaction associated with the peer supervision model:

*We've been able to learn together, figure out what's working for us and what's not working. And when things go wrong, we can sit down and discuss it pretty quickly and easily... We're able to work it through it I think. We have a retreat every year that helps us gather our thoughts and make plans for the next year and assess how this past year went.... So, we're constantly engaging in a review process*.

Consistent with the Knaw Chi Ge Win model, peer supervision is commonly used in multi-disciplinary teams with experienced providers; however this model tends to support established, site-specific norms and may not challenge workers sufficiently. Providers are therefore considering case audits and more direct supervision of new workers.

### Case Management

The mental health nurse case manager, a core team member, provides case management for geriatric clients and clients with SMI. The case manager monitors clients' medications and condition at least once per month, and sometimes as often as once per week. The case manager collaborates with the visiting psychiatrist in person during monthly visits and via fax for the remainder of the month. The psychiatrist informs local primary physicians through progress notes. The case manager also works with community-based paraprofessional workers to enhance acceptance of clients with SMI at the community level, to ensure monitoring, and to coordinate other essential services such as housing or job searching. Further case management has not been implemented due to lack of resources. Priority clients for future case management are high risk clients and those who have multiple re-admissions to the program. The development of a collaborative case management model that suits the Knaw Chi Ge Win model for mental health is important since a cookie cutter approach would not be appropriate for this service environment.

### Strategies that Support Integration of Western and Aboriginal Approaches to Mental Health

We found that the strategies that promote interprofessional collaboration also generally supported an increased understanding between clinical and traditional Aboriginal providers. However, clinical and traditional approaches are based on two distinct medical systems, values sets and philosophies and they are not always easily reconcilable. An in depth understanding of both systems is required for successful integration. The level of integration between clinical and traditional Aboriginal services has progressed significantly within this team from working in a co-operative, multidisciplinary fashion and mainly interacting through referrals which described services in early 2000[33]. Since then, the Knaw Chi Ge Win team developed additional strategies that, as this research suggests, provided the foundation for greater levels of integration between clinical and traditional services including shared intake, case coordination and case collaboration: the development of traditional healing protocols and ongoing capacity building for traditional healing.

### Traditional Aboriginal Healing Protocols

Traditional Aboriginal healing has been practiced for thousands of years in Aboriginal communities, however providing traditional medicine integrated in a clinical setting is a ground breaking practice. Although Aboriginal healers are exempted from governmental regulation in Ontario [34], the health boards decided that guidelines for the provision of traditional healing in a clinical setting based on local Aboriginal culture and values were necessary for successful collaboration. Traditional healing protocols were therefore established based on extensive consultations with Aboriginal community members. As part of this protocol, healers are screened for their area of expertise and their community recognition as an Aboriginal healer and are expected to follow the culturally-based code of conduct[23]. The protocols have arguably served to unveil some of the mystique that traditional medicine often embodies for clinically trained professionals and thus advances integrated practice. As a result, clinicians reported high comfort levels referring to traditional healers and other traditional providers:

I always ask clients within the first or second visit if they would like to see a traditional healer, I always ask them that, I make it a point. (Clinician #1)

I think the Traditional program is very important for the continuum of mental health care. (Clinician #2)

Although many clinicians also revealed that they need to gain a better understanding of traditional healing in order to move towards more profound levels of integration:

We need to be working more with the traditional models... when a person defines that that's the way that they want to go....I think we can, [but] it's a difficult fit, it's like... oil and vinegar, those are two similar concepts. You're trying to fit in something that just is not really well understood. (Clinician #3)

I think with traditional healers we can [develop] more of a mechanism where we could talk about particular clients and do case managements or some sort of case collaboration or shared care...With elders, I think, it would be valuable to discuss more general things like, what's happening in their community, what trends do they see...How's it different then the way they think it could be?.What role can we play... have them understand what we try and do with people...A meeting of the minds with elders from time to time! (Clinician #4)

### Ongoing Capacity Building and Education

In order to sustain collaborative community-based care that integrates both clinical and Aboriginal approaches, extensive education and capacity building targeted at the Knaw Chi Ge Win team as well as community-based workers, clients, and communities as a whole was required. The main areas of capacity building include: (1) to enhance cultural understanding through exposure to traditional Aboriginal teachings, and opportunities to participate in cultural events and traditional medicine practices; (2) ongoing professional development in the area of Aboriginal mental health for professionals and paraprofessional providers; and (3) building capacity with clients and communities to manage their illness and adopt healthy behaviours[26].

## Discussion

### Benefits of Aboriginal Community-based Collaborative Care

Our research shows that the Knaw Chi Ge Win collaborative care model has resulted in several benefits: improved illness care and cultural safety, managed wait times, and reduction in professional isolation.

### Improved Quality of Illness Management

The quality of services has been enhanced for Aboriginal clients in many ways. Although health outcome data were not yet systematically collected, our research suggests that the overall local management of SMI has greatly improved. In-house program statistics show that prior to implementation of this model, 3-4 clients per year had acute care admission to psychiatric hospitals, with frequent recidivism. With the advent of collaborative care, this number was reduced to 0-1 clients per year. Providers explain that clients are more stable and care is more effectively handled locally with this service approach, which they believe explains this reduction in acute care admissions. A mental health worker illustrated why acute admission rates have been reduced with the following case scenario:

*Our approach, it's based upon building capacity in the client to the highest level they can manage themselves...NOT doing it for them... You may walk someone through something the first time and then the second time, they want to do as much as they can. As a result you have individuals who are, managing things a lot more than they ever did in the past... There's a client who is schizophrenic [and], used to be in and out of a psychiatric facility every [few] weeks, back and forth, and back and forth... What's going on here? The psychiatrist believed that the community-based paraprofessional workers could support the client... [but] there was miscommunication there, and unrealistic expectations... So, Knaw Chi Ge Win team members, connected with the long term care program...and decided who was going to do what with the client. There were also housing issues that needed to be looked after, financial issues... the team could connect with the appropriate people [at the community] who could share this load*.

Other indicators of quality in this service environment include the clients' right to choose clinical or traditional approaches without judgment from providers, or receiving care in their Aboriginal language. For example, many geriatric clients who access mental health services prefer to speak their Aboriginal language. Prior to the development of the collaborative services, mental health services were provided by English speaking providers. Now, the team strives to have at least one Aboriginal staff member fluent in Aboriginal language(s) involved in care for geriatric clients. In addition, an estimated 30 - 40 percent of geriatric clients request traditional healing services and information about Aboriginal herbal medicines is openly shared in this service environment. This is particularly significant since research elsewhere shows that the vast majority of clients will conceal the use of traditional medicine from clinical staff[35].

Privacy is another important consideration for many clients who access community-based care in small rural communities, where client anonymity associated with large urban centres does not exist and mental health services are often stigmatized. Clients felt that the services provided by the Knaw Chi Ge Win team were completely confidential. A client explains:

I phone all over for the Knaw Chi Ge Win team. If [my regular worker] isn't there, then I'm looking for [another team member]. I find them! They're more trusted... maybe it's because they don't live here and they're not part of our family they don't know me [personally]. I don't know how I'd feel if I had to go speak to the [paraprofessional] worker in my community if I had a drug and alcohol problem...with, the relationships we have in the community...You can't counsel your own family. (Client #8)

Further evidence of quality services are the high levels of client satisfaction:

"One of the counsellors told me to start keeping a journal of my thoughts, of my feelings so I did that for about a year. When I looked back on it I could see my attitude changing day to day in my writings."(Client #7)

"They helped me control myself in that sense, I guess. Because [before I received services] I couldn't talk to anybody. I was pretty messed up. I guess I... started coming out of my shell and that was a while ago. So now I'm just open and honest. I tell it the way it is and that's just the way I am now. Have my self-esteem, courage. I was scared to do anything, go anywhere, talk to anybody. Be me. So they taught me lots."(Client #11)

"It definitely helped me, even just having someone to talk to, that you could be confident with; that you could speak out and say what you really felt and know it wasn't going anywhere... For me it takes a lot to get my trust after everything that's happened to me in my life. The emotional things I've been through in my life. It's very hard for me to trust somebody, to 100% tell them how I really feel, and [my Knaw Chi Ge Win worker] was that one person I could do that with." (Client #3)

"If I ever needed to talk to anybody or needed help... we have the resources [at Mnaamodzawin] that I can phone...I would phone [the intake worker] right away if it ever came to that. But right now everything is good." (Client #13)

"I think everything was done really well. I was really comfortable talking with the counsellor, and when my husband went to talk to the counsellor too, he thought: "I don't think I'm going to take [mental health services]!", But then at the end of it all he was the one who did most of the talking, he said, he found it really good and it was helpful to him because there was a lot of things that he had to work out and he didn't know how to deal with it. So I think that he really liked the counsellor and so does my daughter." (Client #12)

### Well Managed Wait Times for Mental Health Services

Keeping wait times to a minimum is a priority for the team and clients. Our research revealed that normal response time for urgent care is less than a week, whereas less urgent counseling is normally provided within 3 to 4 weeks. Wait times for traditional healing are longer, often 4 weeks or more, and access to psychiatric services may be several months for new clients. However, these specialized services were not accessible at all prior to the development of integrated services. Due to limited resources, approximately 40 percent of all clients who seek services through Knaw Chi Ge Win are however referred to private providers who operate under the FNIHB short term counseling program.

### Cultural Safety

Cultural awareness, competence, sensitivity and safety have specific meanings that are debated in the academic literature[36]. However these distinctions are less important to clients who seek good, appropriate and respectful care for themselves. To learn about local definition of cultural issues in mental health care we focused on eliciting clients' and providers' personal concepts of appropriate approaches for mental health services for Aboriginal people. Clients and providers generally believed that culturally appropriate care means providing a safe environment for clients to present concerns without judgment from providers. Participants articulated that a cultural focus should go beyond offering traditional healing services. Of great importance to many was provider acceptance of clients' beliefs, religions, backgrounds, and history, and a focus on building on the strengths of Aboriginal people.

This local definition is very much in line with the concept of cultural safety, which originates with the Maori People of New Zealand[37], and also describes a concept that goes beyond cultural competence and focuses on provider self-reflection and understanding of power differentials as well as the central notion that it is the client who defines "safe services"[38] One participant explained this eloquently:

As an Aboriginal person I would say meeting people at their level means culturally competent care. Sometimes people assume that we all believe in traditional [Aboriginal] approaches but that's not necessarily so. A lot of our young people...are enmeshed in the main stream approaches so you have to meet them at their level. Yes, [often] they're very interested in learning about their traditional ways; or they're interested in some mainstream approaches. So you have to meet them...wherever they're at. Also culturally competent care means RE-building (emphasis by participant) their capacity [to heal]... We all have that capacity, it just needs to be RE-built. Versus saying," we need to build it" - No! Everybody has that innate strength in them to take care of themselves and we just need to help them in the right direction. (Participant)

Aboriginal clients consistently described high levels of cultural safety within the Knaw Chi Ge Win team members, regardless of the ethnicity of the provider(s). Clients saw this as a feature of the Knaw Chi Ge Win services which, in their experience, clearly differs from mainstream approaches. Many participants explained that the Knaw Chi Ge Win providers understand Aboriginal issues and are open to Aboriginal world views and healing practices. One client stated:

"Living on the reserve is a different way of life...a different way of thinking. Maybe some needs are different. A lot of people I talked to in the past who were counsellors that hadn't worked for Mnaamodzawin or Noojmowin. - they didn't understand certain things that seems like it's a part of your life when you're on the reserve. It's a different way of thinking. A different way the whole community deals with things. These two [Knaw Chi Ge Win] counsellors understand that; it's not even an issue."(Client #2)

Providers echoed this sentiment and explained that the cultural training they had received had taught them about cultural norms and experiences that could be easily misinterpreted and medicalized by less experienced clinical providers:

The every day experience of people in the communities, how these communities work, we understand that. We understand something about the culture... appreciate the fact that when clients talk about ceremonies, we have an idea what they're talking about, when they talk about spirituality, when they talk about spirits, when they talk about dreams, when they talk about different concepts that are really important in their culture. We understand what they're communicating to us... and what that experience might mean for them... Not that I have a great understanding of it, but, learning bit by bit, the client's cultural world view. We hear about the four directions and the four colours...and the meanings of balance in this world view and that's different than what you're getting in a non-native, euro-centric world view. [As a mental health professional] you got to understand that there are differences and you got to appreciate that. The people who come for the service, they are adhering to that world view to one degree or another and you got to figure out to what degree! (Knaw Chi Ge Win Team Member)

However, clients did express concern about the larger network of service providers in the region, many of whom are perceived as not taking traditional approaches and Aboriginal world views seriously. Consequently, a repeatedly identified issue was the need for more Aboriginal workers at all tiers of service provision. Furthermore, despite the fact that non-Aboriginal core team members are regarded as highly culturally competent, some Aboriginal clients felt much less comfortable with non-Aboriginal than with Aboriginal providers. The clients' life experiences with racism and discrimination appear to be a determining factor. One participant explained this as follows:

*By sending someone to a counsellor who is non-native that puts the playing field where one person is an expert and one is below that - it creates that idea of when you were in school, teachers were non-native, doctors are non-native, everybody is non-native. I don't see that as a way to bring back personal power to a person*. (Participant)

In contrast, a fair number of clients are not comfortable seeking help for mental health issues from community members, and therefore welcome a provider who is not from their community. These clients felt quite comfortable with non-Aboriginal providers as an alternative, while still others professed no preference regarding the ethnicity of their provider within the Knaw Chi Ge Win team. The collaborative care approach allows the team to be responsive to these diverse comfort levels of clients.

### Benefits for Service Providers

Providers see the collaborative practice as a positive and desirable work environment which reduces professional isolation. Providers felt very supported in their work and able to draw on other interdisciplinary team members when necessary, as evidenced in this quote:

Ah, the support!...Basically, the communication is, is just tremendous. I've never worked with a bunch of people where we sit down and talk so much and discuss cases...It's really a benefit not only to everyone as a group to know, how things are going, but, it certainly benefits myself because, they've got the skills that I don't have and what I have I can augment a little bit on their side too. So I think, the shared care model as far as I'm concerned is the only way to go. (Knaw Chi Ge Win Team member)

Clinical as well as traditional service approaches are explicitly respected by all team members. Providers felt that this model had increased their confidence dealing with complex cases, and improved their professional abilities and their ability to ensure cultural safety. New care providers felt that the integration model allows them to work at full capacity soon after they are hired, because existing staff act as mentors during the weekly intake meetings and through informal consultations. The collaborative model has thus contributed to the creation of a relatively stable mental health team in a challenging, resource-scarce service environment.

### Ongoing Challenges

While there are many benefits associated with this model of care, significant challenges still remain to maintain this model of care and to expend the services to a level of care that meets the needs of the community.

### Chronic Under-funding of Aboriginal Mental Health Services

Severe funding constraints for Aboriginal mental health programs make it difficult to recruit and retain experienced qualified professional providers. Funding is often short term and inadequate to attract mental health professionals with sufficient qualifications. The mental health team cannot meet the increasing demands for services (e.g.: 8% increase in 2007). As a result, about 40% of clients must be referred to 'outside' providers for short term counseling under FNIHB's crisis counseling program and this rate will likely increase. There is no collaborative care with these providers once clients are referred out and while outcomes are therefore unknown, the ideal of holistic care is certainly not realized. Additional services in psychiatry, child psychiatry and traditional medicine are needed to meet community needs. Prevention activities for children, youth and parents are also urgently required. In order to expand integration, more time and resources are required for program development and reflection. Providers generally believed there is great potential:

*When I get a chance to listen to what [the healer] has to say, I can hear what his perspective is. People think it's so far apart but I never see it that way, I always think there are so many possible meeting points, when I listen to him speak it's like: Okay! I'm with you on this and this...I think there is all sorts of potential, for more collaboration and more integration if we had the time to work on exactly that... And I think that's true for discussions with elders, you know, I wish we could have some process by which we regularly engaged in that because I'll bet you that there would be more convergence than divergence. That's just the way I see it. (Knaw Chi Ge Win team member)*.

### Recruitment of Mental Health Professionals

Clients value experienced workers and the fact that many of the core team members have been employed with their organizations for many years. However, when positions need to be filled, recruitment of experienced workers is difficult. There is a need for long-term strategies to mentor and recruit Aboriginal students into mental health careers; funds to cover travel costs for student placements are also required.

### Lack of Community Mental Health and Consistent Mental Health Services Data

Reliable mental health status and service data are required to evaluate services and track outcomes. Ongoing systematic data collection is necessary, including: (1) client service data and client satisfaction questionnaires, (2) client outcome data, including changes in client behaviours, symptoms and family situation, (3) local emergency use and hospitalization data in the Manitoulin district, and (4) health status data.

In addition, research is necessary to develop an Aboriginal approach to mental health service evaluation. Existing outcome indicators are based on clinical approaches in mainstream populations and do not take into account Aboriginal understandings of healing or the multi-generational effects of colonization on mental health [39].

## Conclusions

Using a collaborative community-based mental health care approach, the Knaw Chi Ge Win team has developed successful strategies to overcome many barriers to Aboriginal mental health service provision in a rural complex care environment. Their approach offers an efficient use of sparse resources and maximizes their clients' access to specialized services and continuity of care. This approach has also facilitated significant progress in the integration of traditional Aboriginal healing methods with counseling services in a clinical setting. Despite the numerous benefits, additional resources are required to develop further specialized and preventative services to address complex intergenerational community mental health issues. While we do not suggest that the Knaw Chi Ge Win model should be transplanted into Indigenous mental health systems worldwide, the strategies for collaboration we describe are promising practices and may be adapted to other rural and Indigenous systems. Further research into traditional Aboriginal approaches to mental health and their integration with clinical approach is needed to provide a knowledge base to advance integration beyond the regional level.

## Competing interests

The authors declare that they have no competing interests.

## Authors' contributions

MAM participated in the conception, design and coordination of the study, conducted interviews and drafted the manuscript. BE, DG, TG, MS participated in the conception, design of the study and helped to draft the manuscript. LM and MES conducted interviews and helped to draft the manuscript. TLL participated in the data analysis and helped to draft the manuscript. All authors read and approved the final manuscript.
